# Steamed *Panax notoginseng* and its Saponins Inhibit the Migration and Induce the Apoptosis of Neutrophils in a Zebrafish Tail-Fin Amputation Model

**DOI:** 10.3389/fphar.2022.946900

**Published:** 2022-07-07

**Authors:** Yin Xiong, Mahmoud Halima, Xiaoyan Che, Yiming Zhang, Marcel J. M. Schaaf, Minghui Li, Min Gao, Liqun Guo, Yan Huang, Xiuming Cui, Mei Wang

**Affiliations:** ^1^ Faculty of Life Science and Technology, Kunming University of Science and Technology, Kunming, China; ^2^ Institute of Biology Leiden, Leiden University, Leiden, Netherlands; ^3^ Leiden University–European Center for Chinese Medicine and Natural Compounds, Institute of Biology Leiden, Leiden University, Leiden, Netherlands; ^4^ Center for Drug Discovery & Technology Development of Yunnan Traditional Medicine, Kunming, China; ^5^ SU Biomedicine B.V., Leiden, Netherlands

**Keywords:** steamed *Panax notoginseng*, saponin, neutrophil, migration, apoptosis, zebrafish, immune modulation

## Abstract

*Panax notoginseng* (PN) is a Chinese medicinal herb that is traditionally used to treat inflammation and immune-related diseases. Its major active constituents are saponins, the types and levels of which can be changed in the process of steaming. These differences in saponins are causally relevant to the differences in the therapeutic efficacies of raw and steamed PN. In this study, we have prepared the extracts of steamed PN (SPNE) with 70% ethanol and investigated their immunomodulatory effect using a zebrafish tail-fin amputation model. A fingerprint-effect relationship analysis was performed to uncover active constituents of SPNE samples related to the inhibitory effect on neutrophil number. The results showed that SPNE significantly inhibited the neutrophil number at the amputation site of zebrafish larvae. And SPNE extracts steamed at higher temperatures and for longer time periods showed a stronger inhibitory effect. Ginsenosides Rh_1_, Rk_3_, Rh_4_, 20(*S*)-Rg_3_, and 20(*R*)-Rg_3_, of which the levels were increased along with the duration of steaming, were found to be the major active constituents contributing to the neutrophil-inhibiting effect of SPNE. By additionally investigating the number of neutrophils in the entire tail of zebrafish larvae and performing TUNEL assays, we found that the decreased number of neutrophils at the amputation site was due to both the inhibition of their migration and apoptosis-inducing effects of the ginsenosides in SPNE on neutrophils. Among them, Rh_1_ and 20(*R*)-Rg_3_ did not affect the number of neutrophils at the entire tail, suggesting that they only inhibit the migration of neutrophils. In contrast, ginsenosides Rk_3_, Rh_4_, 20(*S*)-Rg_3_, and SPNE did not only inhibit the migration of neutrophils but also promoted neutrophilic cell death. In conclusion, this study sheds light on how SPNE, in particular the ginsenosides it contains, plays a role in immune modulation.

## 1 Introduction

*Panax notoginseng* (PN) (Burk.) F. H. Chen, also named *sanqi*, is a renowned medicinal herb that has been used in Asia to treat inflammation and blood diseases for thousands of years. More than 300 species of Chinese herbal preparations contain PN root and/or rhizome, which are widely applied clinically for disorders such as cardiovascular diseases, atherosclerosis, diabetes, trauma and hemorrhage (Wang et al., 2016; Duan et al., 2017). Saponins are considered to be the major active components of PN, which can be obtained through ethanol extraction (Hu et al., 2018a; Xu et al., 2019). Previous research in our laboratory showed that a high temperature and long steaming time promoted the conversion of saponins in PN, which led to a differentiation in the bioactivities and clinical efficacies between raw and steamed forms of PN (Xiong et al., 2017; Xiong et al., 2019). For example, raw PN (RPN) is better in relieving swelling and easing pain, whereas steamed PN (SPN) shows more tonifying effects such as enhancing immunity and ameliorating anemia. Both of those effects could be related to the immune-modulating activity of these herbal medicines, but the corresponding active components and related mechanisms are still unknown, which hinders the application and further development of this herbal medicine (Kang and Min, 2012; He et al., 2018).

Zebrafish is a widely used animal model that has emerged in recent years as a model system for drug discovery and research of mechanisms underlying multiple disorders, which is utilized as a rapid and high-throughput drug screening system (Wang et al., 2013). The immune system of zebrafish is similar to that of humans, and zebrafish are therefore increasingly used to study diseases related to the immune system, such as inflammation and cancer (Trede et al., 2004). Within only 3 days, zebrafish embryos develop into the larval stage with an immune system consisting of neutrophils and macrophages which orchestrate the innate immune response. Using different transgenic lines in which these cells are fluorescently marked, the activation of the innate immune system can be visualized by imaging the migration of immune cells in a zebrafish tail-fin amputation model (Li et al., 2012). This approach is well suited to study the immunomodulatory activity of the active components of PN. In a previous study (He et al., 2020), we have investigated the immunomodulation by the ginsenoside Rg_1_ using zebrafish larvae as an animal model. And we found that it acted as a selective glucocorticoid receptor agonist with anti-inflammatory action without affecting tissue regeneration.

In the present study, the powdered PN samples were extracted using 70% ethanol and purified to obtain the extracts of PN based on our previously reported methods (Hu et al., 2018a; Hu et al., 2018b). The immunomodulatory effects of these extracts were studied by determining the number of neutrophils migrating towards the wounded site of the zebrafish larval tail after amputation. And beclomethasone (Beclo), a classical glucocorticoid receptor agonist, was used as the positive control. Meanwhile, we developed the HPLC chromatographic fingerprints of PN samples, and investigated the correlation between the effects and fingerprints by using multivariate regression techniques. Major peaks predicted to be correlated with the immunomodulation by PN were then identified to be several ginsenosides, of which the activities were finally verified by pharmacological tests.

## 2 Materials and Methods

### 2.1 Chemicals

The reference standards of ginsenosides Rh_1_, Rk_3_, Rh_4_, 20(*R*)-Rg_3_, and 20(*S*)-Rg_3_ were purchased from Shanghai Yuanye Biotechnology Co., Ltd. (Shanghai, China), with the purity ≧ 98%. Methyl alcohol and acetonitrile (HPLC grade) were purchased from Sigma-Aldrich, Inc. (St. Louis, MO, United States). Ultrapure water was generated with an UPT-I-20T ultrapure water system (Chengdu Ultrapure Technology Inc., Chengdu, China). All other chemicals used were of analytical grade.

### 2.2 Sample Preparation

The preparation of samples was performed as described in previous studies from our laboratory (Hu et al., 2018a; Xiong et al., 2018). PN was obtained from a single batch of root in Yunnan, China (104°077′E, 23°188′N), which had been identified by Prof. Xiuming Cui in Kunming University of Science and Technology. The specimen (No. WSPN15101) has been deposited in Yunnan Key Laboratory of *P. notoginseng*, Kunming University of Science and Technology (Kunming, China), which can be fully validated using http://mpns.kew.org/mpns-portal/?_ga=1.111763972. 1427522246.1459077346. The quality of PN was consistent with the requirements of the Chinese Pharmacopoeia of 2020 edition (Chinese Pharmacopoeia Commission, 2020). SPN was prepared by steaming the crushed RPN in an autoclave (Shanghai, China) for 2, 4, 6, 8, and 10 h at 105°C, 110°C, and 120°C. The steamed powder was dried in a heating-air drying oven at about 45°C to constant weight, then powdered and sieved through a 40-mesh sieve. The powdered PN of 5.0 g was extracted using 50 ml of 70% ethanol at 85°C in a water bath for 1.5 h. After three times of extraction, the ethanol-reflux extracts were combined. Subsequently, the combined extract was centrifuged, filtered, concentrated, and dried to obtain the crude PN extracts. To purify the above crude PN extracts to obtain higher concentrations of saponins, an optimized purification process with macroporous resin was performed, with the concentration of saponin solution of 11.22 mg/ml, loading volume of 4.97 BV, washing volume of 2 BV, ethanol concentration of 70%, and ethanol elution volume of 3.31 BV.

### 2.3 HPLC Analyses

The sample solutions were prepared as described in a previous study (Xiong et al., 2017). Briefly, HPLC analyses were done on an Agilent 1260 series system (Agilent Technologies, Santa Clara, CA, United States) consisting of a G1311B Pump, a G4212B diode array detector, and a G1329B autosampler. A Vision HT C18 column (250 mm × 4.6 mm, 5 μm) was adopted for the analyses. The mobile phase consisted of A (ultra-pure water) and B (acetonitrile). The gradient mode was as follows: 0–20 min, 80% A; 20–45 min, 54% A; 45–55 min, 45% A; 55–60 min, 45% A; 60–65 min, 100% B; 65–70 min, 80% A; 70–90 min, 80% A. The flow rate was set at 1.0 ml/min. The detection wavelength was set at 203 nm, the column temperature at 30°C and the sample volume at 10 μl.

### 2.4 Zebrafish Lines and Maintenance

Zebrafish (*Danio rerio*) were maintained and handled according to the guidelines from the Zebrafish Model Organism Database (http://zfin.org) and in compliance with the directives of the local animal welfare committee of Leiden University. They were exposed to a 14 h light and 10 h dark cycle to maintain circadian rhythmicity. Fertilization was performed by natural spawning at the beginning of the light period. Eggs were collected and raised at 28°C in egg water (60 μg/ml Instant Ocean sea salts and 0.0025% methylene blue). The following transgenic zebrafish lines were used in this study: *Tg*(*mpx:GFP* ^*i114*^ */mpeg1:mCherry-F* ^*umsF001*^) and *Tg*(*mpx:GFP*)^*i114*^ *.*

### 2.5 Tail Fin Amputation and Drug Treatments

Three-day-old zebrafish larvae were utilized for the tail fin amputation experiments. In each experiment, 20 larvae were used in each treatment group. The administration included a vehicle (Veh) group treated with 0.01% DMSO as the negative control, a group treated with Beclo of 25 μM as the positive control, and groups subjected to the following treatments: RPN powder at 50 μg/ml; RPN extract (RPNE) at 50 μg/ml; SPN powder at 50 μg/ml; SPN extract (SPNE) at 30 μg/ml; and three concentrations (at 30, 60, and 90 μM) each for Rh_1_, Rk_3_, Rh_4_, 20(*R*)-Rg_3_, and 20(*S*)-Rg_3_. In a pilot experiment, no toxicity on the survival of zebrafish larvae was observed for any group (data not shown). All groups were pretreated with Veh/Beclo/experimental treatment for 2 h before tail fin amputation, and received the same treatment for 4 h after the amputation. Next, larvae were anesthetized in egg water containing 0.02% buffered amino benzoic acid ethyl ester (Sigma-Aldrich Chemie N.V., Zwijndrecht, Netherlands). Larvae were placed on petri dishes coated with 2% agarose under a Leica M165C stereomicroscope, and the tail fins were partly amputated using a 1 mm sapphire blade. For quantification of leukocyte migration, larvae were fixed overnight in 4% paraformaldehyde at 4°C.

### 2.6 Visualization and Quantification of Neutrophils

Imaging of the *Tg*(*mpx:GFP* ^*i114*^ */mpeg1:mCherry-F* ^*umsF001*^) larvae was performed utilizing a LeicaMZ16FA fluorescence stereomicroscope supported by LAS 3.7 software. The neutrophils were detected based on the green fluorescence of their GFP lalabed. To quantify the number neutrophils recruited to the wounded area, the cells in a defined area of the tail as well as in the entire tail were counted manually.

### 2.7 Partial Least Squares Regression

PLSR is performed to find the inner relationship between the independent variables (*X*) and dependent variables (*Y*), which are simultaneously modeled by taking into account *X* variance, and the covariance between *X* and *Y* (Martens and Naes, 1991). In our study, the *X* matrix is composed of the enhanced fingerprints, and the *Y* vector is constructed with the relative inhibition rate (*RI*_*i*_%) calculated by Eq. 1. Then, *X* and *Y* are decomposed in a product of another two matrices of scores and loadings, as described by Eqs 2, 3:

$$RI_{i}\%=\left(\left(n_{\max}-n_{i}\right)/n_{\max}\right)\times 100\%$$ (1)

$$X=TP^{T}+E$$ (2)

$$Y=UQ^{T}+F$$ (3)

Where $n_{\max}$ is the maximal number of migrated neutrophils at the amputation site found in any of the treated groups, $n_{i}$ is the number of migrated neutrophils treated by PN sample i , and $RI_{i}\%$ is the reference inhibition rate of neutrophils determined for PN sample i . $TP^{T}$ approximates the chromatographic data and $UQ^{T}$ to the true *Y* values; notice that the relationship between the T and U scores is a summary of the relationship between X and Y . The terms E and F from the equations are error matrices. Hence, the PLSR algorithm attempts to find latent variables that maximize the amount of variation explained in *X* that is relevant for predicting *Y*; i.e., capture variance and achieve correlation (Brereton, 2007).

### 2.8 Terminal Deoxynucleotidyl Transferase dUTP Nick-End Labeling Assay

Apoptotic cell death was detected by the Transferase dUTP Nick-End Labeling (TUNEL) assay with the *In Situ* Cell Death Detection Kit, Fluorescein (Roche Diagnostics GmbH, Mannheim, Germany). Briefly, *Tg*(*mpx:GFP*)^*i114*^ zebrafish larvae at 3 days post-fertilization (dpf) were euthanized, fixed, dehydrated, digested, and post-fixed following the manufacturer’s protocol. The neutrophils and apoptotic neutrophils were labeled with green and red fluorescence, respectively. Then, the quantification of migrated neutrophils was performed as described above.

### 2.9 Statistical Analyses

All data are expressed as means ± standard deviation. IBM SPSS Statistics 20.0 software (IBM North America, New York, NY, United States) and GraphPad Prism 6 (GraphPad Software, San Diego, CA, United States) was applied to carry out the two-tailed unpaired *t*-test and one-way ANOVA. Umetrics SIMCA-P 11.5 software (Sartorius Stedim Biotech GmbH, Goettingen, Germany) was applied for PLSR analysis. A value of *p* < 0.05 was considered significant, and a value of *p* < 0.01 was considered highly significant.

## 3 Results

### 3.1 HPLC Analyses

**FIGURE 1:**
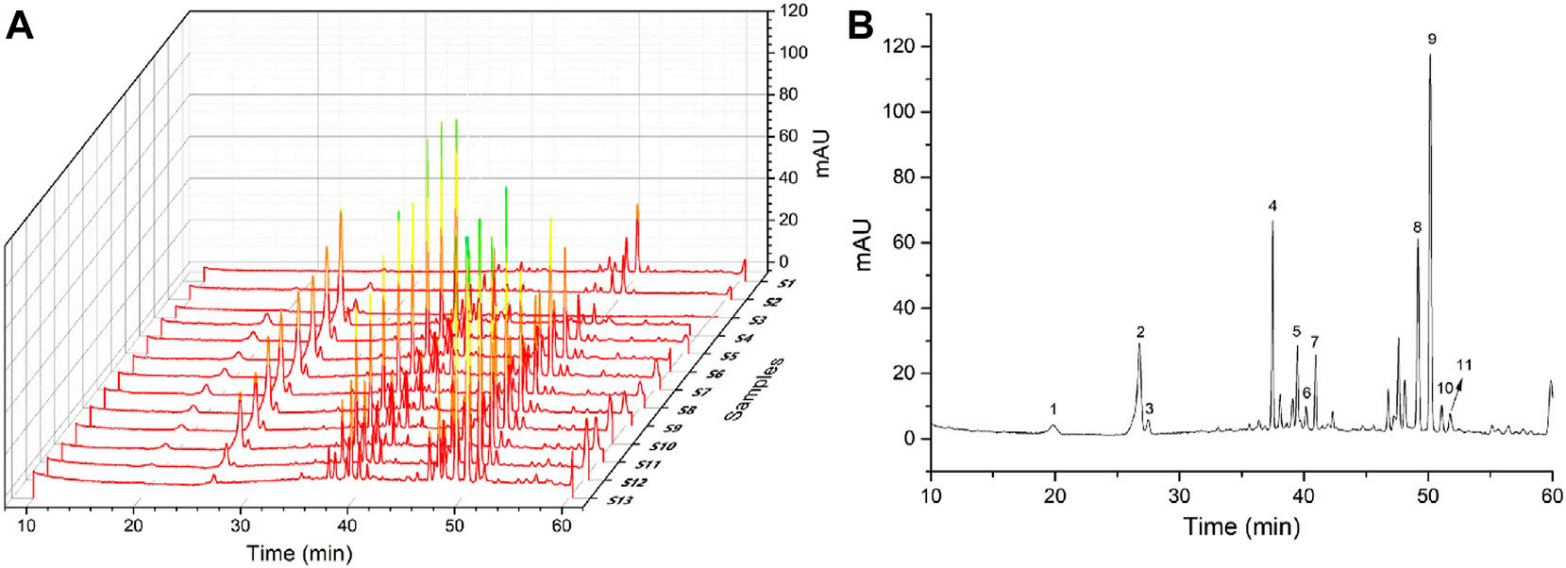
**(A)** Chemical chromatograms of 13 batches of PN samples and **(B)** 11 selected peaks marked on the chromatogram of S10.

The information and HPLC fingerprints of 13 batches of PN samples, i.e., powder and extract of RPN and SPN prepared under different steaming time periods and temperatures, are shown in Figure 1. These fingerprints indicate a distinct difference in the chemical composition between raw and steamed samples, as well as a much higher level of major peaks in the extract compared to powder of the same steaming condition. Consecutive peaks with good segregation and large areas were determined as the common peaks of PN samples. As a result, 11 peaks were selected by comparing their ultraviolet spectra and HPLC retention time, which were then used for further analysis of the fingerprint-effect relationship. The areas of these 11 peaks in the 13 batches of PN samples are listed in Table 1. Along with the duration of steaming time and rise of temperature, the area and height of major peaks (peaks 1–4, and 7) were decreased gradually, while other peaks (peaks 5, 6, 8–11) were increased or formed. The peak area was defined as 0 when a peak was absent in a chromatogram. The coefficients of variance for all common peaks were higher than 43.85%, which is due to the diversity in the levels of components contained in samples under different processing conditions. Besides, the areas and height of PNE were much larger than the powder, suggesting a higher level of active constituents and possibly stronger bioeffects.

**TABLE 1:** Peak areas of eleven common peaks in PN and PNE samples.

No.	Sample^a^	Peak number										
		1	2	3	4	5	6	7	8	9	10	11
S1	RPN	38.24	191.17	0.00	113.23	0.00	0.00	31.63	0.00	0.00	0.00	0.00
S2	SPN120°C-2 h	0.00	0.00	0.00	28.59	44.53	16.01	12.39	182.32	360.13	32.97	0.00
S3	SPN120°C-6 h	0.00	0.00	0.00	65.43	30.23	0.00	24.24	100.48	193.62	0.00	0.00
S4	RPNE	246.46	1533.16	249.52	866.13	90.59	15.00	285.18	50.82	100.80	55.62	0.00
S5	SPNE105°C-2 h	329.95	1432.35	261.00	885.48	81.51	21.68	279.02	120.20	246.94	66.24	0.00
S6	SPNE105°C-4 h	263.97	1380.61	174.94	1152.93	133.25	60.53	400.48	364.69	710.97	125.74	28.17
S7	SPNE105°C-6 h	281.04	1363.17	151.30	910.12	126.03	75.15	305.61	512.12	1019.41	139.13	39.10
S8	SPNE110°C-2 h	257.58	1342.24	0.00	867.53	98.59	39.23	269.37	298.42	602.43	88.43	23.05
S9	SPNE110°C-4 h	213.23	1180.66	187.59	827.83	110.62	49.81	273.76	459.39	910.72	110.56	29.82
S10	SPNE110°C-6 h	192.64	908.16	0.00	679.83	111.31	90.79	241.45	773.01	1560.13	172.65	57.94
S11	SPNE120°C-2 h	189.05	901.29	144.66	716.86	114.82	84.19	235.62	664.81	1322.47	139.68	42.01
S12	SPNE120°C-4 h	0.00	415.83	0.00	140.49	121.31	160.20	122.98	1334.54	2620.14	225.45	98.12
S13	SPNE120°C-6 h	0.00	0.00	0.00	142.15	128.92	191.16	64.80	1466.95	2913.80	254.35	105.24
	C.V.^b^ (%)	78.71	71.71	113.27	68.16	43.85	92.07	62.77	92.72	92.46	70.23	106.95

a RPN, raw *Panax notoginseng*; SPN, steamed *Panax notoginseng*; RPNE, raw *Panax notoginseng* extract; SPNE, steamed *Panax notoginseng* extract.

b C.V. (%) = δ/μ × 100; δ is the standard deviation, μ is the average value of peak area.

### 3.2 Inhibition of Zebrafish Neutrophils by *Panax notoginseng* Samples

**FIGURE 2:**
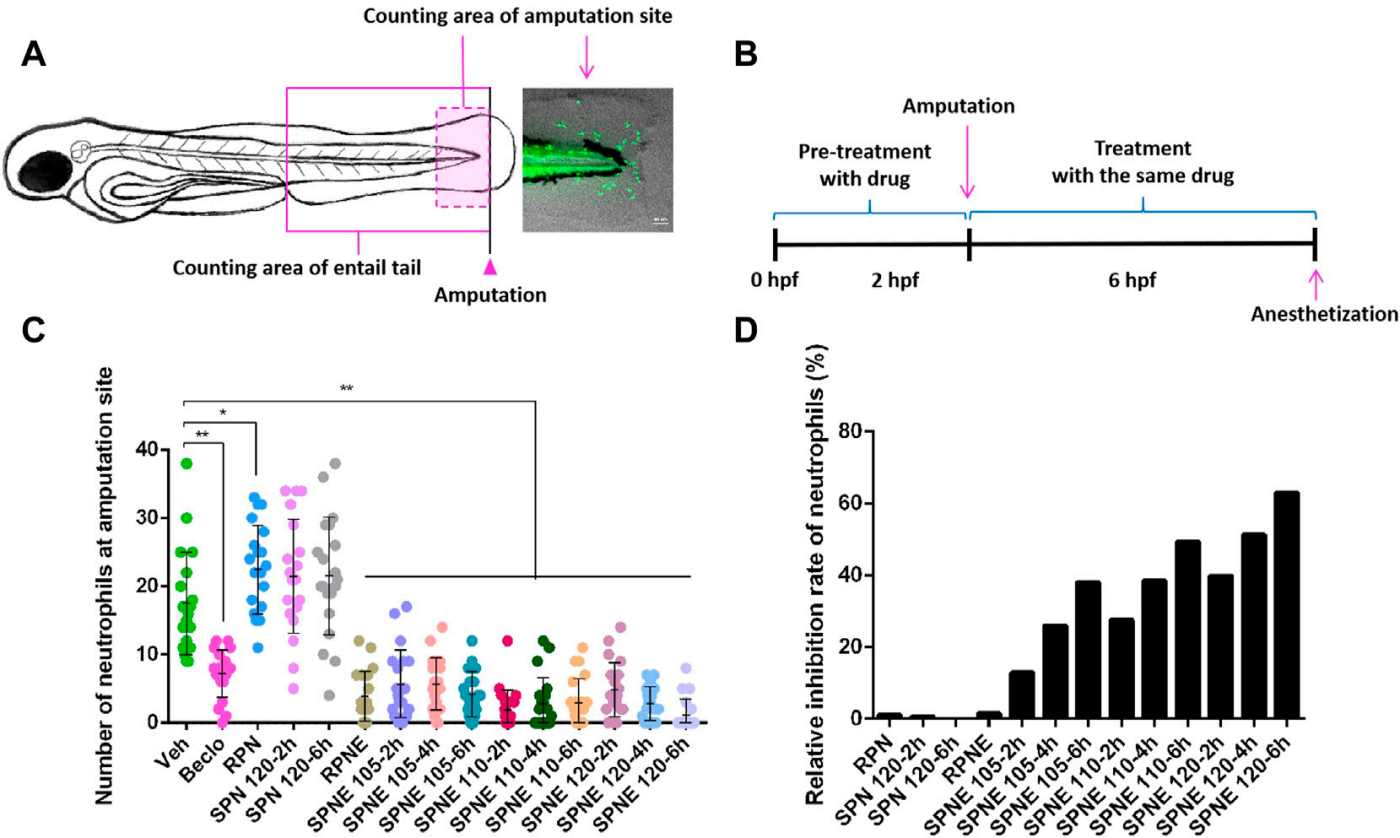
The effect of different PN samples on neutrophil recruitment in the zebrafish tail fin amputation assay. **(A)** The counting areas for the quantification of neutrophils, and a representative fluorescence microscopy image of amputation-induced migration of neutrophils in zebrafish larvae at 3 dpf. **(B)** Schematic of the drug treatment in the zebrafish tail fin amputation experiments. **(C)** The number of neutrophils at the amputation site at 4 h after amputation upon treatment with different PN samples. **(D)** The relative inhibition rate of neutrophils after treating with different PN samples. Hpf, hours post-fertilization; Veh, vehicle; Beclo, beclomethasone; RPN, raw *Panax notoginseng*; RPNE, raw *Panax notoginseng* extract; SPN, steamed *Panax notoginseng*; SPNE, steamed *Panax notoginseng* extract. *p < 0.05 and **p < 0.01.

The transgenic *Tg*(*mpx:GFP* ^*i114*^ */mpeg1:mCherry-F* ^*umsF001*^) zebrafish line in which neutrophils are labeled by GFP, enables the analysis of the behaviour of neutrophils *in vivo*. By visualizing the neutrophils that have migrated to the wounded area in the zebrafish tail fin amputation model, compounds that affect neutrophil behaviour and affect the immunomodulation response can be screened (Renshaw et al., 2006; Wang et al., 2013; Chatzopoulou et al., 2016). In this study, the neutrophil number at the injury region was determined in zebrafish larvae at 3 days post-fertilization (dpf) at 4 h after amputation (Figure 2A). After pre-treatment with different drugs for 2 h, the amputation was performed, following a 4-h treatment with the same drug (Figure 2B). The results showed that RPN and SPN powder did not significantly inhibit the number of neutrophils at the amputation site. In contrast, the number of neutrophils at the amputation site was decreased significantly upon treatment with Beclo or PNE. SPNE samples steamed at a higher temperature and for longer periods of time showed a stronger inhibiting effect (compared to the Veh group) on neutrophil number at the amputation site (Figure 2C) and higher relative inhibition rate (Figure 2D). To identify the constituents of SPNE responsible for the inhibition on neutrophils, a multivariate data analysis was subsequently performed to correlate the chemical data.

### 3.3 Uncovering Active Constituents by Multivariate Data Analysis

#### 3.3.1 Prediction

**FIGURE 3:**
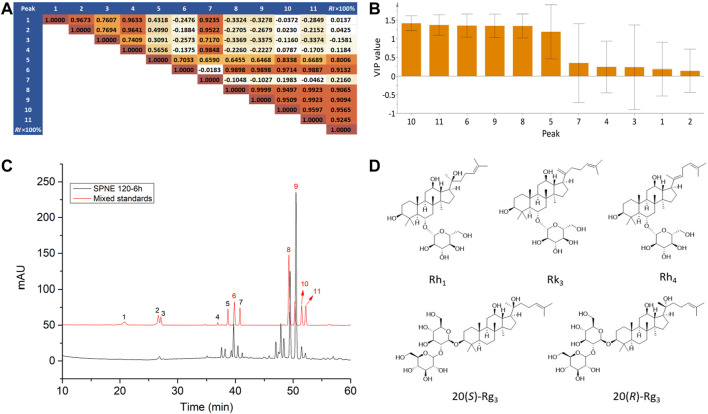
Uncovering active constituents by multivariate data analysis. **(A)** Correlation matrix and **(B)** VIP values of peak areas correlating with the relative inhibition rates of PN samples. **(C)** The chromatograms of the mixed standards solution and SPNE sample steamed at 120°C for 6 h. Peaks 6, 8, 9,10 and 11 correspond to ginsenosides Rh_1_, Rk_3_, Rh_4_, 20(*S*)-Rg_3_, and 20(*R*)-Rg_3_, respectively. **(D)** Structures of the ginsenosides Rh_1_, Rk_3_, Rh_4_, 20(*S*)-Rg_3_, and 20(*R*)-Rg_3_.

Currently, methods for uncovering active constituents of herbal medicines that are used for treating diseases mainly rely on retrospective analysis. However, this method depends on a large consumption of manpower and material resources. To address this issue, the relative inhibition rates of neutrophils at the amputation site were determined for 13 batches of PN samples, and they were linked with the peak areas in the corresponding chemical fingerprints to construct a “fingerprint-effect relationship” by PLSR. By analyzing the relationship model and weight coefficients, active constituents could be preliminarily predicted despite changes in the peaks and their areas in the chromatogram. Since the total number of samples (13) was small and the prediction for new samples was not our first concern, no division was made into a calibration set to build a PLSR model and a test set to validate the predictive properties. PLSR models were built from the normalized data matrix *X* containing the 13 PN fingerprints and the response matrix *Y* of the reference inhibition rate of neutrophils at the amputation site. For the model, two principle components were determined, accounting for an explained variance of 94.9% for the *X* variable, 92.0% for the *Y* variable, and a predictive ability (*Q* ^*2*^) of 84.8%, indicating that the obtained model was excellent. As shown in the coefficient matrix in Figure 3A, the correlation coefficients between peaks 6, 8, 9, 10, and 11 and the reference inhibition rate were higher than 0.9, indicating that the area of these peaks had a very high level of correlation with the inhibiting effect of PN. Besides, the importance of the *X*-variables for the model could be summarized by variable importance for the projection (VIP) values (usually with a threshold > 1.0), which showed that these VIP values of peaks 6, 8, 9, 10, and 11 were all higher than 1.0 (Figure 3B). Thus, constituents corresponding to peaks 6, 8, 9, 10, and 11 were considered to be related to the inhibition of the neutrophil migration by different PN samples.

By comparing the chromatograms of PN samples to that of the mixture of reference substances (Figure 3C), peaks 6, 8, 9, 10, and 11 were identified as ginsenosides Rh_1_, Rk_3_, Rh_4_, 20(*S*)-Rg_3_, and 20(*R*)-Rg_3_ respectively (Figure 3D). As shown in Table 1 and Figure 1, the areas of peaks 6, 8, 9, 10, and 11 of SPNE samples were increased along with the steaming time and elevation of steaming temperature. Thus, the ginsenosides Rh_1_, Rk_3_, Rh_4_, 20(*S*)-Rg_3_, and 20(*R*)-Rg_3_, might play a major role in the neutrophil-inhibiting effect of SPNE.

#### 3.3.2 Verification

**FIGURE 4:**
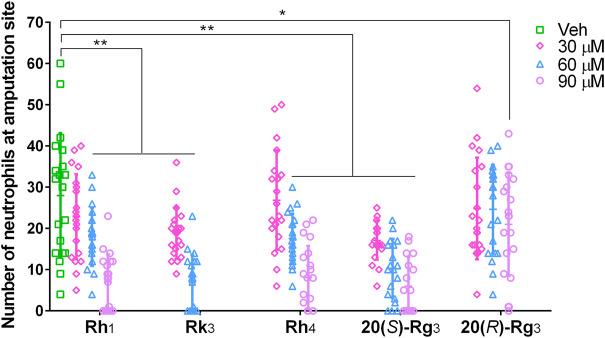
Inhibiting effects of different doses of ginsenosides Rh_1_, Rk_3_, Rh_4_, 20(*S*)-Rg_3_, and 20(*R*)-Rg_3_ on neutrophil recruitment at the amputation site of zebrafish larvae. Veh, vehicle. *p < 0.05 and **p < 0.01.

In order to verify the predicted result, the effects of ginsenosides Rh_1_, Rk_3_, Rh_4_, 20(*S*)-Rg_3_, and 20(*R*)-Rg_3_ on neutrophil number were evaluated using the zebrafish tail fin amputation model. According to the results shown in Figure 4, the five ginsenosides all decreased the number of neutrophils at the amputation site in a dose-dependent way. Rh_1_ and Rh_4_ at 60 and 90 μM, Rk_3_ at 30 and 60 μM, and 20(*S*)-Rg_3_ at 30, 60 and 90 μM showed a highly significant inhibitory effect (*p* < 0.01). The ginsenoside 20(*R*)-Rg_3_ at 90 μM also significantly decreased the neutrophil number (*p* < 0.05). Since Rk_3_ at 90 μM appeared to be lethal to the zebrafish larvae, this concentration was excluded in the effective doses of Rk_3_ in this case. Taking the results above together, we verified that ginsenosides Rh_1_, Rk_3_, Rh_4_, 20(*S*)-Rg_3_, and 20(*R*)-Rg_3_ were the major active constituents of SPNE to inhibit neutrophil number in the zebrafish tail fin amputation model.

### 3.4 Steamed *Panax notoginseng* Extract and Its Major Ginsenosides Inhibit the Migration and Also Induce Apoptosis of Neutrophils

**FIGURE 5:**
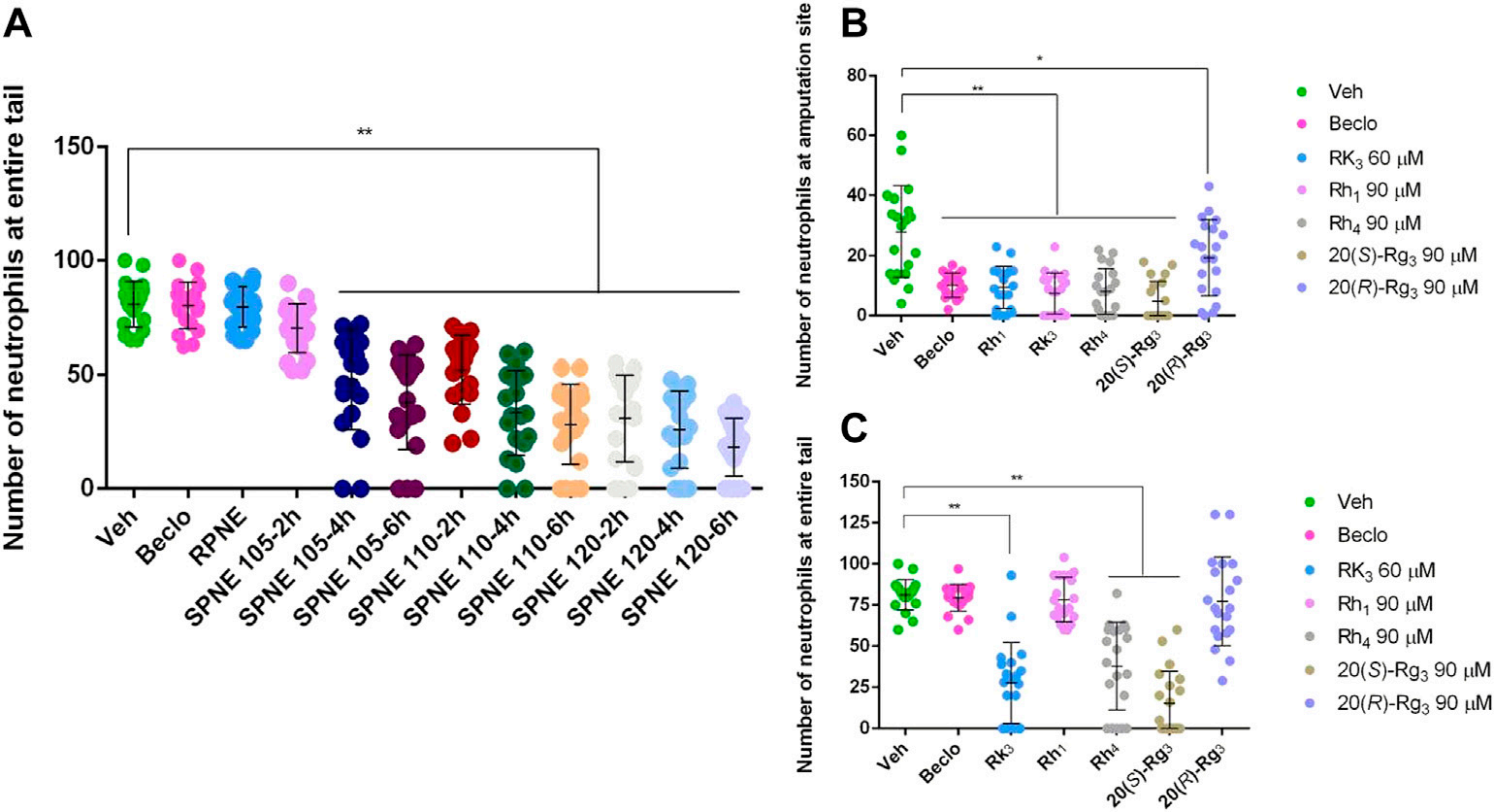
The effect of SPNE and its major ginsenosides on neutrophil migration. **(A)** The number of neutrophils in the entire tail of amputated zebrafish larvae after treatment with different PNE samples. **(B,C)** The number of neutrophils at the amputation site **(B)** and in the entire tail **(C)** of amputated zebrafish larvae after treatment with ginsenosides Rh_1_, Rk_3_, Rh_4_, 20(*S*)-Rg_3_, and 20(*R*)-Rg_3_. Beclo, beclomethasone; RPNE, raw *Panax notoginseng* extract; SPNE, steamed *Panax notoginseng* extract; Veh, vehicle. *p < 0.05 and **p < 0.01.

Neutrophils migrate rapidly to sites of inflammation. The resolution of this inflammatory response can be achieved by either reversing the migration of neutrophils or by the well-characterized process of neutrophil apoptosis (Renshaw et al., 2006; Loynes et al., 2010). To better understand the mechanism underlying the decreased number of neutrophils in the zebrafish tail fin amputation model upon treatment with SPNE and its major active ginsenosides, we investigated the number of neutrophils in the entire tail after amputation. Interestingly, no inhibition on the total number of neutrophils in the entire tail was observed after treatment with Beclo, RPNE, and SPNE steamed at the lowest temperature of 105°C and at the shortest time of 2 h (Figure 5A). This, combined with the result in Figure 2C, indicates that they mainly inhibit the migration of neutrophils to the injury site. In contrast, SPNE prepared at a higher temperature or for a longer period of time all induced a significant decrease in the total number of neutrophils in the entire tail (Figure 5A). This indicates that the decreased number of neutrophils at the amputation site observed after these treatments (Figure 2D) could be due to both the migration-inhibiting and elimination effects of SPNE on neutrophils.

To further explore if the previously identified five active constituents of SPNE showed similar effects on the total number of neutrophils in the tail, we compared the effects of ginsenosides Rh_1_, Rk_3_, Rh_4_, 20(*S*)-Rg_3_, and 20(*R*)-Rg_3_ on the number of neutrophils at the amputation site and in the entire tail of zebrafish larvae. As shown in Figures 5B,C, all five ginsenosides at the tested dose significantly inhibited the number of neutrophils at the amputation site, confirming the results shown in Figure 4. However, Rh_1_ and 20(*R*)-Rg_3_ did not affect the number of neutrophils in the entire tail, suggesting they only inhibit the migration of neutrophils to the amputation site. In contrast, ginsenosides Rk_3_, Rh_4_, and 20(*S*)-Rg_3_ induced a significant decrease in the number of neutrophils in the entire tail, indicating that these three compounds not only inhibit the migration of neutrophils but also promote the death of neutrophils.

**FIGURE 6:**
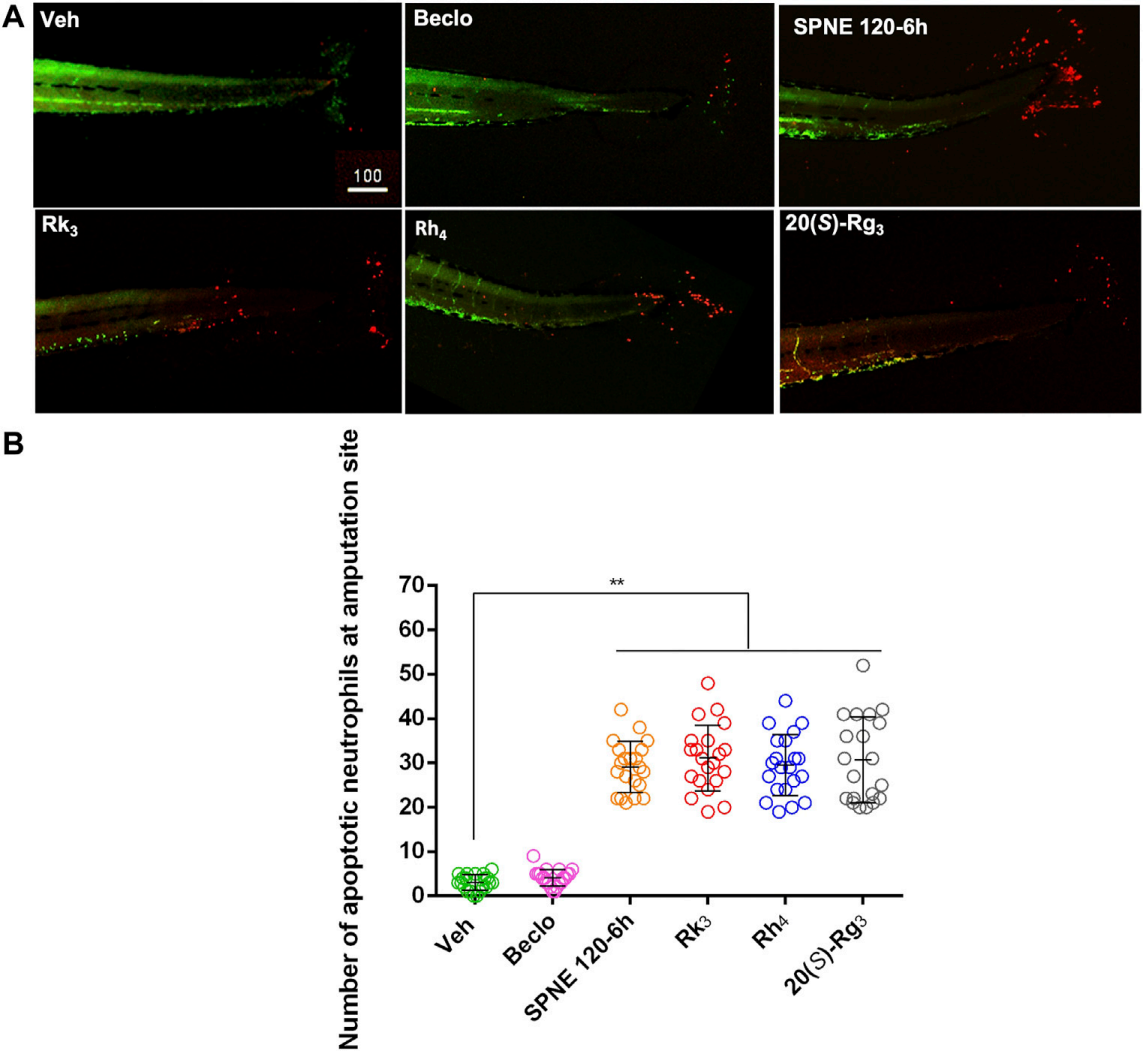
The effect of SPNE and its major ginsenosides on apoptotic neutrophils. **(A)** Apoptotic neutrophils labeled by TUNEL staining in amputated tails of zebrafish larvae. **(B)** The number of apoptotic neutrophils at the amputation site after treating with ginsenosides Rh_1_, Rk_3_, Rh_4_, 20(*S*)-Rg_3_, and 20(*R*)-Rg_3_. Beclo, beclomethasone; SPNE, steamed *Panax notoginseng* extract; Veh, vehicle. *p < 0.05 and **p < 0.01.

Based on the above results, we hypothesized that the death of neutrophils could be due to apoptosis. This hypothesis was tested using TUNEL staining of the zebrafish larvae, which detects DNA fragmentation that is characteristic for (but not specific to) apoptotic cells. Images resulting from the application of this assay to larvae from the *Tg*(*mpx:GFP*)^*i114*^ show the neutrophils labeled in green and apoptotic neutrophils labeled in red (Figure 6A). These images showed that SPNE, ginsenosides Rk_3_, Rh_4_, and 20(*S*)-Rg_3_ triggered the apoptosis of neutrophils mainly at the amputation site of zebrafish larvae (Figure 6A). Compared to the Veh group, the numbers of apoptotic neutrophils were significantly increased by treatments with SPNE, and the ginsenosides Rk_3_, Rh_4_, and 20(*S*)-Rg_3_ (Figure 6B).

## 4 Discussion

Both raw and steamed forms of PN have been used in traditional Chinese medicine to regulate the immune system and treat diseases related to inflammation. Dammarane-type saponins are considered to be the major active constituents of PN, which could be enriched by the ethanol extraction process (Sun et al., 2016; Hu et al., 2018b). The chemical structures of saponins are often changed during the steaming process (Wang et al., 2013). As shown in Figure 1, the areas of major peaks in raw samples were decreased and some other new peaks were produced along with the rise of steaming time and temperature. Such variation in the chemical composition contributes to the difference in the pharmacological effects and clinical efficacies between raw and steamed forms (Xiong et al., 2019). This was illustrated in our study by the observation that SPNE could inhibit the migration of neutrophils and promote their apoptosis, whereas RPNE only impacted the migration (Figures 2, 5).

Neutrophils constitute about 40%–60% of circulating white blood cells in the human body and are highly evolved for host defense through phagocytosis, degranulation, and the formation of reactive oxygen species and neutrophil extracellular traps (Wang et al., 2013). In the meantime, the uncontrolled neutrophilic activity and continued recruitment of neutrophils to inflammatory sites can result in persistent inflammation and exacerbate chronic human diseases related to the immune system (Gernez et al., 2010). Amputation of the tail fin of zebrafish larvae induces the migration of neutrophils towards the wounded site, which enables studying anti-inflammatory drug effects in an *in vivo* vertebrate animal model (Renshaw et al., 2006; Chatzopoulou et al., 2016).

To uncover the specific constituents responsible for the inhibitory effect on neutrophil migration, the multivariate data analysis of PLSR was performed to analyze the “fingerprint-effect relationship” of PN samples. Compared with traditional extraction and separation approaches, this method has several advantages such as being less time- and solvent-consuming, low operating costs, and little pollution to the environment, and has therefore been widely applied to discover active compounds in complex mixtures of herbal medicines (Xu et al., 2014; Feng et al., 2020). Based on the results presented in Figures 3, 4, ginsenosides Rh_1_, Rk_3_, Rh_4_, 20(*S*)-Rg_3_, and 20(*R*)-Rg_3_ were predicted and verified to be the active ones involved in the effect on the number of neutrophils at the amputation site. These ginsenosides were also the major constituents of SPNE.

Combined with our previous results (Xiong et al., 2017; Xiong et al., 2019), notoginsenoside R_1_, ginsenosides Re, Rb_1_, Rg_1_ and Rd were major constituents in raw PN samples. During the steaming process, the hydrolyzation of xylosyl at C-6 of notoginsenoside R_1_ and rhamnosyl at C-6 of ginsenoside Re produced Rg_1_. The further hydrolysis of the glucosyl at C-20 of Rg_1_ yielded Rh_1_ which then formed Rh_4_ and Rk_3_ through dehydration at C-20. Ginsenoside Rb_1_ could be hydrolyzed at the glucosyl of C-20 to yield ginsenoside Rd. Similarly, the hydrolysation of the glucosyl at C-20 of Rd produced Rg_3_ (Wang et al., 2012). This transformation indicated the change of major peaks in the chromatograms of PN samples during the steaming. The levels of ginsenosides Rh_1_, Rk_3_, Rh_4_, 20(*S*)-Rg_3_, and 20(*R*)-Rg_3_ in SPNE were elevated along with the increase of steaming time and temperature, which explained why SPNE processed for longer time periods and at higher temperatures exhibited stronger inhibition on the migration of neutrophils as well as the pro-apoptosis effect on neutrophils.

SPN is traditionally used as a tonic to attenuate the syndrome of “blood deficiency” and help patients to recover from chronic disease in traditional Chinese medicine. Patients or animals with this syndrome often suffer from impaired hematopoietic function, peripheral blood pancytopenia, hypofunction of internal organs, malnutrition, or even hemolysis which promote inflammatory reactions (Zhang et al., 2014; Ji et al., 2017). Activated by inflammatory stimuli, circulating neutrophils are recruited to the injury or infectious sites as the first responders during an innate immune response, of which the retention, however, could lead to tissue damage and even develop to other complications. Therefore, the resolution of neutrophil-mediated inflammation by reversing the migration and inducing the apoptosis of neutrophils could be one of the mechanisms of drugs to alleviate anemia (Da Guarda et al., 2017; Mooney et al., 2018). In our previous studies, SPNE and its saponins were verified to show an anti-anemia effect by reversing the decrease of bloods cells in mice with blood deficiency syndrome (Zhang et al., 2019). The treatments of Rk_3_ and 20(*S*)-Rg_3_ of certain doses could reverse the decreased levels of heme and ferrochelatase, of which the abnormal synthesis can lead to anemia. In the presence of high level of heme, the neutrophil death could be accelerated by antioxidant reagents released by red blood cells (Luo and Loison, 2008). This might explain how SPNE and its ginsenosides played the role in the treatment of anemia by promoting the apoptosis of over retentive neutrophils. One thing should be noted that the effect of a ginsenoside on neutrophil was previously shown in our group to be mediated by the glucocorticoid receptor (He et al., 2020). But no effects of activation of this receptor has been found on apoptosis, which suggests that the effect of SPNE on apoptosis is most likely not mediated by this receptor but through activation of another pathway. Besides, the inappropriate delay of neutrophil death within tissues has been often implicated in a variety of inflammatory and immunological diseases. Neutrophil apoptosis in patients with both infective and non-infective insults-elicited systemic inflammatory response syndrome decreases significantly (Jimenez, 1997; Melley et al., 2005). With the anti-neutrophil treatment, the damage to the lung and liver could be both attenuated (Mercer-Jones et al., 1997). Therefore, SPNE and its ginsenosides showed the potential to be developed as drugs with neutrophil-inhibiting effect for the treatment of immune diseases.

## 5 Conclusion

By using the zebrafish larval tail fin amputation model, the effects on neutrophil recruitment of SPNE obtained using different steaming conditions were investigated in the present study. Combined with the chemical analysis and fingerprint-effect relationship of PN samples, ginsenosides Rh_1_, Rk_3_, Rh_4_, 20(*S*)-Rg_3_, and 20(*R*)-Rg_3_ were found to be the active ones correlated to the effect of SPNE on neutrophils. Among them, Rh_1_ and 20(*R*)-Rg_3_ only inhibited the migration of neutrophils to the amputation site, whereas Rk_3_, Rh_4_, and 20(*S*)-Rg_3_ could also promote the apoptosis of neutrophils. The results shed light on how SPNE and its ginsenosides impact immunity and treat related diseases.

## Data Availability

The original contributions presented in the study are included in the article/Supplementary Material, further inquiries can be directed to the corresponding authors.

## Abbreviations

Beclo, beclomethasone; dpf, days post-fertilization; Hpf, hours post-fertilization; PLSR, Partial least squares regression; PN, *Panax notoginseng*; RPN, raw *Panax notoginseng*; RPNE, raw *Panax notoginseng* extrac; SPN, steamed *Panax notoginseng*; SPNE, steamed *Panax notoginseng* extract; Veh, vehicle
